# Cost effectiveness of adherence to IDSA/ATS guidelines in elderly patients hospitalized for Community-Aquired Pneumonia

**DOI:** 10.1186/s12911-016-0270-y

**Published:** 2016-03-15

**Authors:** Michael E. Egger, John A. Myers, Forest W. Arnold, Leigh Ann Pass, Julio A. Ramirez, Guy N. Brock

**Affiliations:** The Hiram C. Polk, Jr., MD Department of Surgery, University of Louisville, School of Medicine, Louisville, KY USA; Present Affiliation: Department of Surgical Oncology, The University of Texas MD Anderson Cancer Center, Houston, TX USA; Department of Pediatrics, University of Louisville, School of Medicine, Louisville, KY USA; Department of Medicine, Division of Infectious Diseases, University of Louisville, School of Medicine, Louisville, KY USA; Pharmacy Department, University of Louisville Health Care, Louisville, KY USA; Department of Bioinformatics and Biostatistics, University of Louisville, School of Public Health, Louisville, KY USA; Present Affiliation: Department of Biomedical Informatics, The Ohio State University, College of Medicine, Columbus, OH USA

**Keywords:** Community acquired pneumonia, Multi-state model, Markov model, Costeffectiveness, Length of hospital stay, Time to clinical stability, In-hospital mortality

## Abstract

**Background:**

Adherence to guidelines for the treatment of hospitalized elderly patients with community-acquired pneumonia (CAP) has been associated with improved clinical outcomes. This study evaluated the cost-effectiveness of adherence to guidelines for the treatment of CAP in an elderly hospitalized patient cohort.

**Methods:**

Data from an international, multicenter observational study for patients age 65 years or older hospitalized with CAP from 2001 to 2007 were used to estimate transition probabilities for a multi-state Markov model traversing multiple health states during hospitalization for CAP. Empiric antibiotic therapy was classified as adherent, over-treated, and under-treated according to 2007 Infectious Disease Society of America/American Thoracic Society IDSA/ATS guidelines. Utilities were estimated from an expert panel of active clinicians. Costs were estimated from a tertiary referral hospital and adjusted for inflation to 2013 US dollars. Costs, utilities, and transition probabilities were all modeled using probability distributions to handle their inherit uncertainty. Cost-effectiveness analysis was based on the first 14 days of hospitalization. Patients admitted to the intensive care unit (ICU) were analyzed separately from those admitted to the ward. Sensitivity analyses with regards to time frame (out to 30 days hospitalization), cost estimates, and willingness to pay values were performed.

**Results:**

The model parameters were estimated using data from 1635 patients (1438 admitted to the ward and 197 admitted to the ICU). For the ward model, adherence to antibiotic guidelines was the dominant strategy and associated with lower costs (−$1379 and −$799) and improved quality of life compared to over- and under-treatment. In the ICU model, however, adherence to guidelines was associated with greater costs (+$13,854 and + $3461 vs. over- and under-treatment, respectively) and lower quality of life. Acceptance rates across the willingness to pay ranges evaluated were 42–48 % for guideline adherence on the ward and 61–64 % for over-treatment on the ICU. Results were robust over sensitivity analyses concerning cost and utility estimates.

**Conclusions:**

While adherence to antibiotic guidelines was the most cost-effective strategy for elderly patients hospitalized with CAP and admitted to the ward, in the ICU over-treatment of patients relative to the guidelines was the most cost-effective strategy.

**Electronic supplementary material:**

The online version of this article (doi:10.1186/s12911-016-0270-y) contains supplementary material, which is available to authorized users.

## Background

Pneumonia is one of the leading causes of death in the United States, and a leading cause of hospitalizations is community-acquired pneumonia (CAP) [1, 2]. Hospitalizations are increasing for infectious disease-related problems in the elderly, particularly for pneumonia [3, 4]. For these reasons, the treatment of elderly patients hospitalized with CAP is a major component of health care spending in America and an important public health issue.

Clinical practice guidelines have been developed to guide antibiotic choice in hospitalized CAP by the Infectious Diseases Society of America and the American Thoracic Society (IDSA/ATS) [5]. Clinical practice guidelines are becoming more common across multiple medical disciplines. They are generally formed from evidence-based reviews by experts in their field and promulgated by national or international professional societies in an effort to disseminate best practices and improve patient outcomes. There is evidence to suggest that adherence to guidelines may improve mortality and time to clinical stability (TCS) in CAP [6, 7]. Adherence to the 2007 IDSA/ATS guidelines improves mortality, length of stay (LOS), and TCS in elderly patients hospitalized with CAP [8].

Cost effectiveness research will be a critical part of healthcare spending and intervention evaluation in the future of American healthcare. It is incumbent upon practitioners to not only deliver medical care with good clinical outcomes, but also to consider those interventions that most efficiently use scarce healthcare resources. The cost effectiveness of practice guideline adherence has been evaluated and found to be cost-effective in a variety of medical conditions [9–12]. The cost effectiveness of adherence to antibiotic guidelines in CAP has only been evaluated in a limited number of studies [6, 13, 14]. This study was performed in an effort to determine the cost-effectiveness of adherence to IDSA/ATS antibiotic guidelines for the treatment of CAP in hospitalized elderly patients (age ≥ 65).

## Methods

### Data source

This study is a retrospective study utilizing patient and outcome data from the Community Acquired Pneumonia Organization (CAPO) International Study Cohort, which is an international, multicenter observational study of patients hospitalized with CAP [15]. Data were abstracted from the database from June 1, 2001 to January 1, 2007 for all patients age 65 years or older and included data from 43 centers in 12 countries including North America, South America, Europe, Africa, and Southeast Asia (see Arnold et al. [8] for details concerning the patient cohort used in this study and the ‘Availability of Data and Materials’ section). The study was approved by the Human Subject Protection Program Institutional Review Board at the University of Louisville. Additional approval was obtained from the local internal review board for each participating hospital. Patient consent was waived due to the retrospective and observational study design. The initial antibiotic regimen for each patient was evaluated and categorized as adherent, over-treated, or under-treated according to the 2007 IDSA/ATS guidelines as previously described [5, 8]. Patients were stratified into ICU or ward admission status based on their initial admission orders. A patient was considered clinically stable on the day they were switched to oral antibiotics from intravenous antibiotics; ATS criteria for switching to oral therapy were used [16]. A small number of patients were admitted with oral antibiotics as their initial therapy; these patients were considered clinically stable upon admission. Baseline characteristics of the cohorts of interest were compared using chi-square or Fisher exact for categorical data, as appropriate, medians were compared with Kruska-Wallis test, and probabilities compared using Gray’s test for difference between cumulative incidence curves [17].

### Markov model

**Fig. 1:**
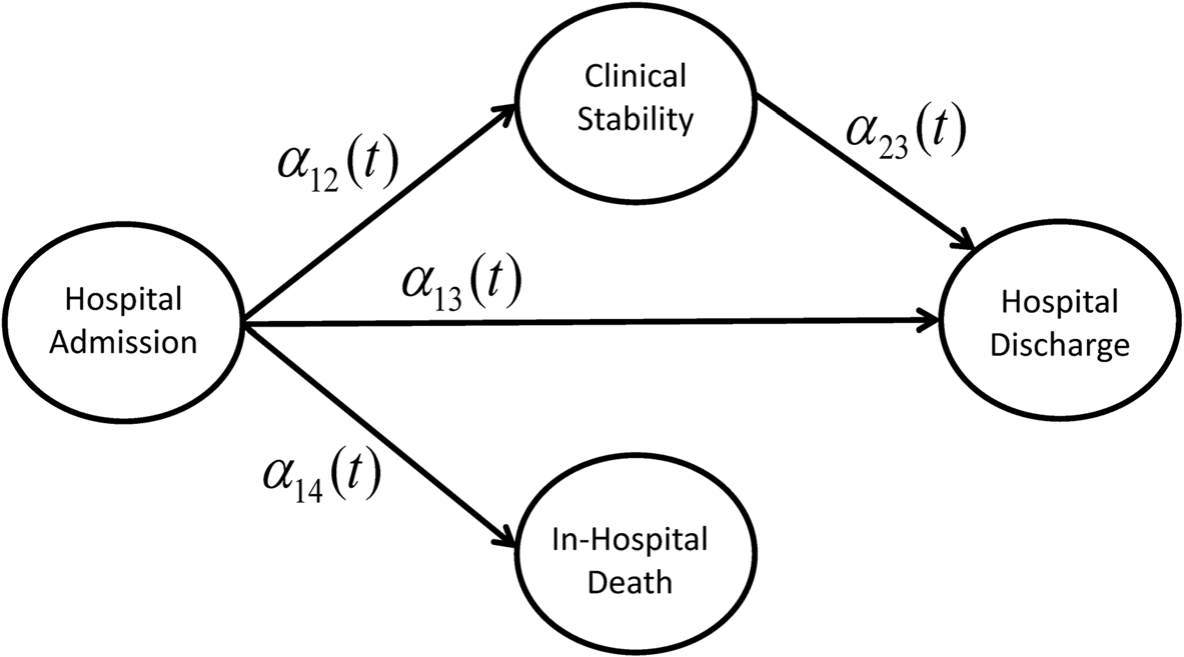
Schematic of multi-state model. Multi-state Markov model of four possible states during hospitalization for community-acquired pneumonia. For each possible transition the $\alpha ij$ ($t$) represent the cause-specific transition hazard from state *i* to state *j*

A 14-cycle Markov model was constructed modeling the four possible states in which a patient could exist during the 14 day observation period (Fig. 1). Each cycle is a 24 h period during the patient’s hospitalization. Patients admitted to the hospital (state 1) remained in this initial state until they either reached clinical stability (state 2), were discharged (state 3), or died in the hospital (state 4). Patients who reached clinical stability remained in that state until they were subsequently discharged. Patients initially admitted to the ICU were assumed to remain in the ICU until they died or reached clinically stability, after which they were transferred to the ward. Prior to reaching clinically stability patients were on intravenous medication, after which they were placed on oral medications.

Conditional transition probabilities for each possible transition from state *i* to state *j* (see Fig. 1) at time *t* ∈ [1, 14] were estimated based on the cause-specific transition hazards *α*_*ij*_ (*t*). Non-parametric estimates of the transition hazards were based on the number of individuals making the *i* → *j* transition at time *t* divided by the number of individuals in state *i* just prior to time *t* (i.e., the number ‘at-risk’ of making the *i* → *j* transition). Non-parametric estimates of the conditional transition probabilities were then obtained using the Aalen-Johansen estimator with the R package *msSurv* [18, 19]. Parametric estimates were obtained by first fitting time-dependent Cox regression models to each of the possible transition hazards outlined in Fig. 1. These estimates of the transition hazards were then used to obtain estimated transition probabilities for an individual with a given covariate profile using the R package *mstate* [20]. The covariates (in addition to adherence to antibiotic guidelines) used in the parametric models were disease severity (pneumonia severity index (PSI) risk class ≥4 vs. <4), presence of multilobar pneumonia, pleural effusion, altered mental status, tachypnea (respiratory rate > 30 breaths/min), hypotension (systolic blood pressure < 90 mm Hg), receipt of antimicrobials within 8 h of admission, pneumococcal vaccination, blood cultures obtained within 24 h of admission, and whether oxygen assessment was done [21]. All covariates were dichotomous, and the parametric transition probabilities were based on the most frequently occurring value for each covariate. Daily transition probabilities (the probability of making an *i* → *j* transition from time *t* to *t* + 1) were obtained from both parametric and non-parametric estimates and compared to ensure validity. The daily transition probabilities from the parametric multi-state model were modeled as continuous beta distributions in the Markov model based on the estimates and standard errors from the parametric model.

The antibiotic decision making was modeled using three comparators: Adherent, Over-treated, and Under-treated [8]. The multi-state Markov process followed the decision making nodes. ICU admissions and ward admissions were analyzed in two separate models. Cost-effectiveness analyses, probabilistic sensitivity analyses, and a Monte Carlo microsimulation with 10,000 patients were performed. For conciseness only results from transition probability estimates based on the parametric models are reported, however substantive conclusions based on the non-parametric estimates did not differ. “Adherent” was considered the base case comparator, against which the over- and under-treated groups were compared.

### Cost and utility estimations

Our model required cost and utility estimations for the following unique states: ICU admission status (over-treated, adherent, and under-treated), ward admission status (over-treated, adherent, and under-treated), ward clinically stable (over-treated, adherent, and under-treated), discharge status, and death. Costs were adjusted according the US consumer price index to 2013 US dollars. Average daily hospital costs for a day in the ICU were dynamically modeled, with mean daily costs ranging from $5132 on ICU day one to $3825 for day eight and thereafter (c.f. medical costs reported in Table 4 in Dasta et al. [22], adjusted to 2013 US dollars). The daily ICU costs were modeled with a gamma probability distribution with parameters κ and θ estimated from reported standard deviations of cost data by Dasta et al. [22] Mean daily ward costs for treatment of CAP were estimated to be a static cost of $1060 per day (c.f. Table 3, column ‘Simple Pneumonia’ in Kaplan et al. [23]). Daily ward costs, like ICU costs, were modeled with a gamma probability distribution with parameters based on standard deviation estimates reported by Kaplan et al. [23] The initial antibiotic regimen of each cohort was retrieved from the CAPO database. Daily antibiotic costs were estimated based on average wholesale prices (year 2013 value) of each antibiotic obtained from the University of Louisville Hospital (Additional file 1). The daily cost of each respective antibiotic regimen in our database was calculated and averaged for our unique states described above. Admission status antibiotic regimens were intravenous, while patients in clinical stability by definition were on oral medications. Patients initially admitted to the ICU were assumed to transfer to the ward upon reaching clinical stability. The cost associated with each state was the sum of the hospital charges for the location of treatment (ICU or ward) and daily estimated antibiotic costs.

Utility estimations were obtained based on a survey (Additional file 2) administered to an expert panel survey of six CAPO investigators. Each expert was polled independently. While the executive director of CAPO assisted in convening the expert panel, he was not involved in the eliciting of utilities and was blinded to specific utility assessments. The demographics of the expert panel survey were as follows: 50 % male; one Fellow, four Assistant Professors, and one Professor; mean age 43.5; mean years of clinical practice experience was 18. All respondents were currently practicing in the US. Each survey respondent was asked to estimate the utility associated with the following states in the context of a standard gamble: ICU admission, ward admission, and ward clinically stable. Utility was defined as an estimate of relative quality of life on a scale of 0 to 1, with 0 being death and 1 being at home. It was assumed that the utility of each state would be independent of the antibiotic adherence classification. The utility estimates were modeled with a beta distribution, with shape parameters based on the standard deviation of the expert panel survey results.

Probabilistic sensitivity analyses were performed for the hospitals costs and utility estimates, since they were modeled as probability distributions. Sensitivity analyses of the fixed daily drug cost estimates were estimated in a 3-way analysis across ranges $1 to $100 in 20 intervals. Strategy dominance was evaluated across willingness to pay ranges from $0 to $1000, in 20 intervals. This range was based on the generally used threshold of willingness to pay of $100,000/QALY. Since our model cycles corresponded to days, rather than years, a willingness to pay range of 0 to $1000 per quality-adjusted life day corresponds to 0 to $365,000 per QALY [24].

TreeAge Pro 2015 (Cambridge, MA) software was used for all cost-effectiveness analyses.

## Results

### Model parameters

There were 1635 patients identified age ≥ 65 whose initial antibiotic regimens could be classified; 1438 of these patients were admitted to the ward and 197 were admitted to the ICU. Patient demographics for the ICU and Ward patients according to antibiotic regimen are summarized in Table 1, while the number and percentage of patients on various antibiotic regimens is given in Table 2.

**Table 1:** Summary of Demographic and Clinical Parameters

		Adherent Ward *n* = 877 ICU *n* = 98	Over-treated Ward *n* = 167 ICU *n* = 28	Under-treated Ward *n* = 394 ICU *n* = 71	p-value
Male Gender	Ward *n* = 1438	544 (62.0 %)	101 (60.5 %)	217 (55.1 %)	0.064^a^
	ICU *n* = 197	58 (59.2 %)	25 (89.3 %)	37 (52.1 %)	0.0026^a^
Risk Class	Ward *n* = 1438				0.0002^a^
	1	0	0	1 (0.3 %)	
	2	45 (5.1 %)	10 (6.0 %)	11 (2.8 %)	
	3	206 (23.5 %)	23 (13.8 %)	76 (19.3 %)	
	4	462 (52.7 %)	82 (49.1 %)	196 (49.8 %)	
	5	164 (18.7 %)	52 (31.1 %)	110 (27.9 %)	
	ICU *n* = 197				0.56^b^
	1	0	0	0	
	2	0	1 (3.6 %)	0	
	3	13 (13.3 %)	2 (7.1 %)	8 (11.3 %)	
	4	43 (43.9 %)	12 (42.9 %)	28 (39.4 %))	
	5	42 (42.9 %)	13 (46.4 %)	35 (49.3 %)	
Nursing Home	Ward *n* = 1438	61 (7.0 %)	27 (16.2 %)	44 (11.2 %)	0.0002^a^
	ICU *n* = 197	4 (4.1 %)	3 (10.7 %)	5 (7.0 %)	0.39^b^
Cancer	Ward *n* = 1438	85 (9.7 %)	24 (14.4 %)	60 (15.2 %)	0.0096^a^
	ICU *n* = 197	9 (9.2 %)	4 (14.3 %)	7 (9.9 %)	0.723^a^
CHF	Ward *n* = 1438	231 (26.3 %)	43 (25.8 %)	104 (26.4 %)	0.99^a^
	ICU *n* = 197	45 (45.9 %)	8 (28.6 %)	35 (49.3 %)	0.16^a^
Stroke	Ward *n* = 1438	186 (21.2 %)	36 (21.6 %)	131 (33.3 %)	<0.0001^a^
	ICU *n* = 197	11 (11.2 %)	0	18 (25.4 %)	0.0023^b^
COPD	Ward *n* = 1438	321 (36.6 %)	66 (39.5 %)	116 (29.4 %)	0.0198^a^
	ICU *n* = 197	40 (40.8 %)	9 (32.1 %	25 (35.2 %)	0.62^a^
Diabetes	Ward *n* = 1438	195 (22.2 %)	44 (26.4 %)	82 (20.8 %)	0.35^a^
	ICU *n* = 197	27 (27.6 %)	7 (25.0 %)	7 (9.9 %)	0.0169^a^
Liver Disease	Ward *n* = 1438	25 (2.9 %)	4 (2.4 %)	12 (3.1 %)	0.91^a^
	ICU *n* = 197	3 (3.1 %)	2 (7.1 %)	0	0.72^b^
Renal Disease	Ward *n* = 1438	121 (13.8 %)	27 (16.2 %)	57 (14.5 %)	0.72^a^
	ICU *n* = 197	18 (18.4 %)	3 (10.7 %)	8 (11.3 %)	0.36^a^
Age	Ward	79	79	80	0.065^c^
	ICU	75	73	79	0.0097^c^
PSI Score	Ward	104	116	109.5	<0.0001^c^
	ICU	123.5	126.5	130	0.6133^c^
Antibiotic Timing	Ward	5	4.5	4.5	0.081^c^
	ICU	6	3.5	5	0.15^c^
Discharge probability (14 day)^e^	Ward	0.77	0.62	0.68	<0.001^d^
	ICU	0.48	0.61	0.40	0.12^d^
Clinical stability probability (7 day)^e^	Ward	0.75	0.57	0.60	<0.001^d^
	ICU	0.40	0.57	0.34	0.027^d^
In-hospital mortality (14 day)^e^	Ward	0.06	0.14	0.13	<0.001^d^
	ICU	0.14	0.18	0.26	0.04^d^

^a^ Chi-square

^b^ Fisher exact test

^c^ Medians compared with Kruskal-Wallis test

^d^ Gray’s test for differences between cumulative incidence curves

^e^ Reported discharge, clinical stability, and in-hospital mortality probabilities are all unadjusted or marginal probabilities

**Table 2:** Number and Proportion of Patients in Antibiotic Groups

	Ward (*n* = 1438)	ICU (*n* = 197)
Regimen	Patients, No. (%)	Patients, No. (%)
Adherent	877 (61 %)	98 (50 %)
β-lactam + macrolide	500 (35 %)	71 (36 %)
β-lactam + macrolide + vancomycin	0 (0 %)	1 (<1 %)
Quinolone	374 (26 %)	13 (7 %)
Quinolone + vancomycin	2 (<1 %)	0 (0 %)
Quinolone + β-lactam	0 (0 %)	12 (6 %)
Quinolone + β-lactam + vancomycin	0 (0 %)	1 (<1 %)
Other	1 (<1 %)	0 (0 %)
Undertreated	394 (27 %)	71 (36 %)
β-lactam	301 (21 %)	42 (21 %)
β-lactam + other^a^	50 (3 %)	15 (8 %)
β-lactam (antipseudomonal) + macrolide	19 (1 %)	8 (4 %)
Macrolide	15 (1 %)	1 (<1 %)
Other	9 (1 %)	5 (3 %)
Overtreated	167 (12 %)	28 (14 %)
β-lactam (antipseudomonal) + macrolide	19 (1 %)	2 (1 %)
β-lactam + macrolide + quinolone or other	49 (3 %)	17 (9 %)
Quinolone + macrolide	8 (1 %)	0 (0 %)
Quinolone + β-lactam +/- other^b^	64 (4 %)	4 (2 %)
Quinolone + other^a^	24 (2 %)	3 (2 %)
Macrolide + other^c^	2 (<1 %)	1 (<1 %)
Other	1 (<1 %)	1 (<1 %)

^a^ other = other than a macrolide

^b^ The +/- here indicates that another antibiotic (other than vancomycin) may or may not have been prescribed

^c^ other = other than a β-lactam

The estimated daily transition probabilities for both the parametric and non-parametric multi-state models are given in Additional file 3 (ward) and Additional file 4 (ICU). The lines represent the probability of making a transition from one state to another on sequential days. The parametric model estimates were based on the covariate profile corresponding to the most frequent value for each covariate. For the ward and ICU models these were PSI risk class ≥4, absence of multilobar pneumonia, pleural effusion, altered mental status, tachypnea, and hypotension, antimicrobials not received within 8 h of admission, no pneumococcal vaccination, and both blood cultures obtained within 24 h of admission and oxygen assessment done. For the ward model the parametric and non-parametric estimates agree fairly closely, with the parametric estimates appearing smoother in the over-treated case. For the ICU model again there is fairly good agreement between the parametric and non-parametric estimates, though the parametric model generally had higher estimated transition probabilities to clinical stability/discharge status and lower transition probabilities to in-hospital mortality. Note that for both ICU and ward models once an absorbing state (discharge and in-hospital death) is reached the probability to remain in that state is one.

**Fig. 2:**
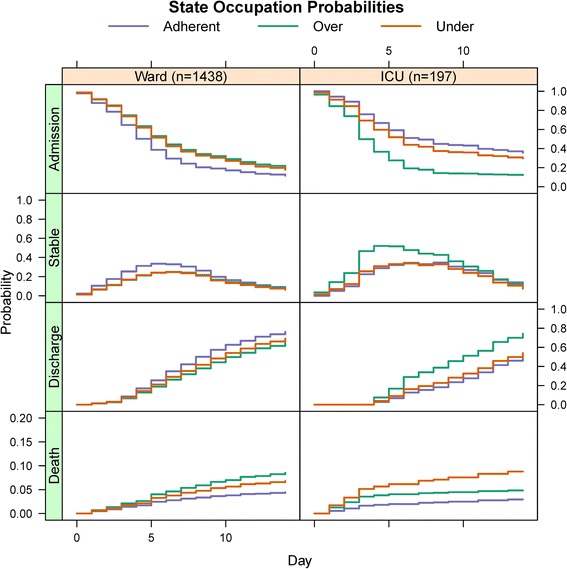
State occupation probabilities of multi-state model. State occupation probabilities in the multi-state Markov model by antibiotic regimen, stratified by admission status (ICU or ward). Each stage corresponds to a single hospital day. Scale for y-axis for in-hospital mortality (death) is from 0.0 to 0.2 to better illustrate separation between the different antibiotic strategies

**Fig. 3:**
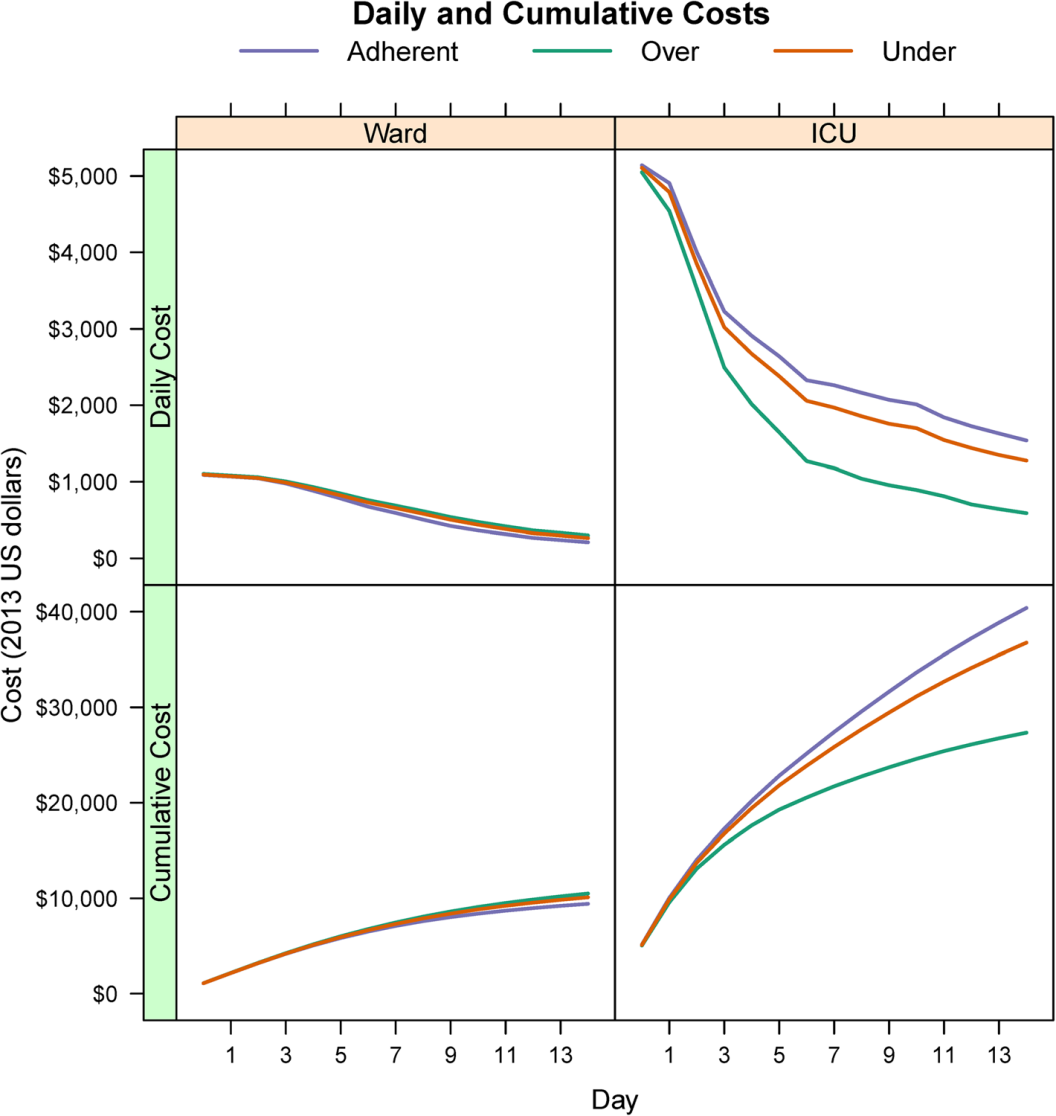
Daily and cumulative cost estimates. Per stage (daily) and cumulative cost estimates according to antibiotic strategies, stratified by ICU or ward admission status

**Fig. 4:**
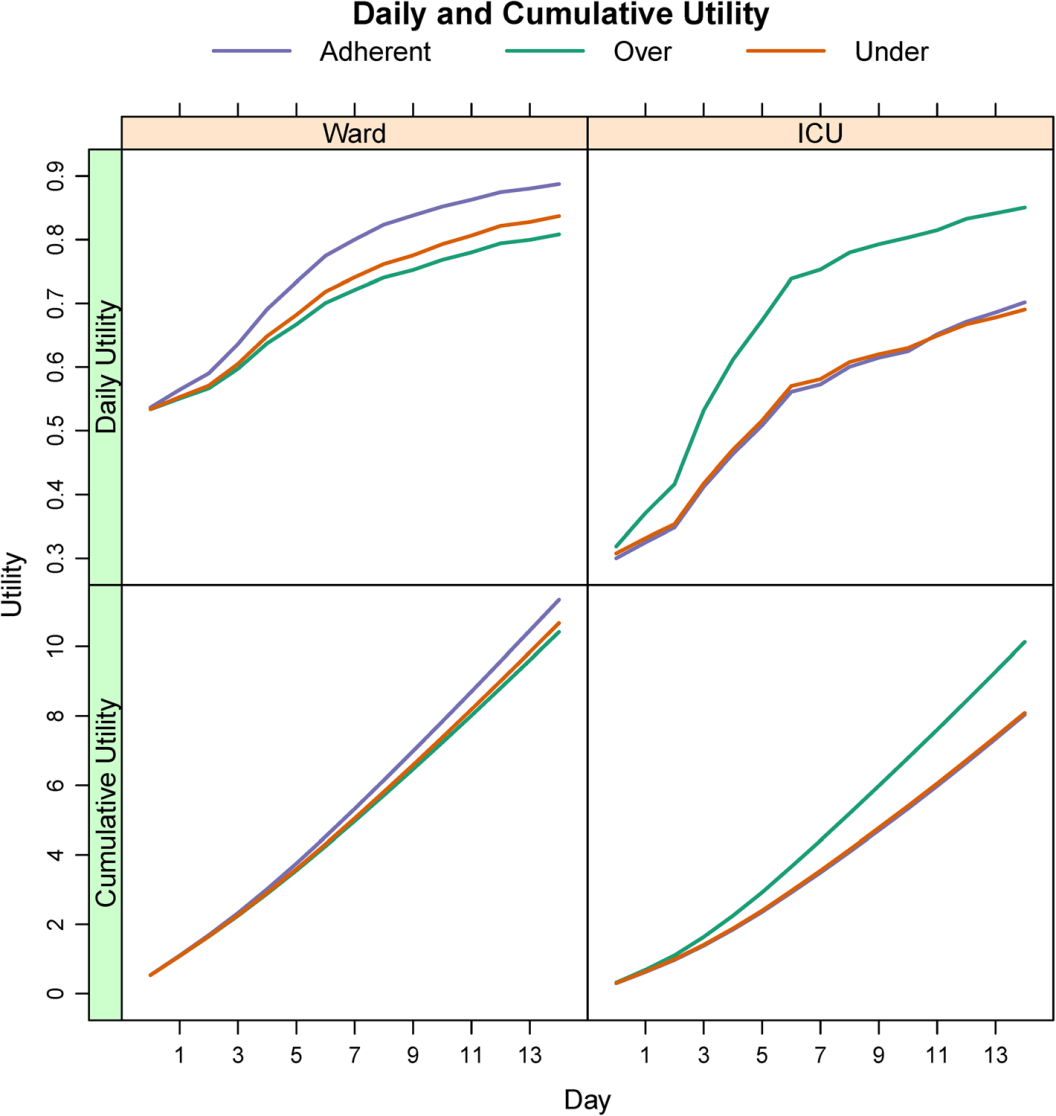
Daily and cumulative utility estimates. Per stage (daily) and cumulative utility estimates according to antibiotic strategies, stratified by ICU or ward admission status

The daily transition probabilities from the parametric model based on the given covariate profile were used to build the Markov model for analysis. The proportions of patients occupying the four unique states in the Markov model over time are represented in Fig. 2. Daily cost and utility estimates for each state are given in Table 3. Per stage and cumulative cost estimates for both models are presented in Fig. 3, demonstrating decreased daily and cumulative costs for the adherent cohort relative to the over- and under-treated cohorts in the ward model. However, in the ICU model the over-treated cohort had decreased costs compared to the adherent and under-treated cohorts. Per stage and cumulative utility estimates are presented in Fig. 4, demonstrating increased utility in the adherent strategy in the ward model and the over-treated strategy for the ICU model.

**Table 3:** Cost and Utility Estimations^a^

	ICU	Ward
Daily Intravenous Antibiotic Cost ($)^b^		
Adherent	41.82	30.61
Over-Treated	64.85	43.91
Under-Treated	36.57	34.97
Daily Oral Antibiotic Cost ($)^b^		
Adherent	12.58	14.75
Over-Treated	20.50	23.10
Under-Treated	9.28	9.53
Daily Hospital Cost ($)^c^	5132 (4767) – 3825 (2658)	1060 (1141)
Utility^d^		
Admission Status	0.3 (0.063)	0.53 (0.10)
Clinically Stable	0.82 (0.13)	0.82 (0.13)
Discharge	1	1
Dead	0	0

^a^ Reported as mean (standard deviation)

^b^ Daily intravenous and oral antibiotic costs were estimated based on 2013 US wholesale prices from University of Louisville Hospital (Additional file 1)

^c^ Daily hospitalization costs were estimated from Dasta et al. [22] for ICU and Kaplan et al. [23] for ward

^d^ Daily utility estimates were based on a questionnaire administered to a panel of six experts (Additional file 2)

### Base case analysis

The cost effectiveness analysis results for the ward model are summarized in Table 4. The adherent strategy was the dominant cost-effective decision in this set of patients. Patients treated with an antibiotic regimen that was adherent to the recommendations of the 2007 IDSA/ATS guidelines experienced lower costs and increased utility compared to patients treated with an over- or under-treatment regimen. On average, total hospitalization costs were $1379 and $799 less in the adherent group compared to the over- and under-treated groups, respectively.

**Table 4:** Cost effectiveness results in the ward model of hospitalized community-acquired pneumonia in the elderly according to antibiotic regimen. Increment cost, utility, and cost/utility ratio were estimated relative to adherent as the base comparator

	Adherent	Over-Treated	Under-Treated
Cost ($)	10,156 (4665 – 17,585)	11,535 (5339 – 20,179)	10,954 (5159-19,190)
Incremental Cost ($)		+1379 (−8418 – 11,448)	+799 (−8875 – 10,940)
Utility	11.2 (8.9 – 12.5)	10.1 (7.2 – 12.1)	10.5 (7.9 – 12.1)
Incremental Utility		−1.0 (−4.2−1.9)	−0.7 (−3.6–2.0)

Estimates presented as means (2.5^th^-97.5^th^ percentile). Costs are in 2013 US dollars

The cost effectiveness analysis results for the ICU model are summarized in Table 5. The over-treatment strategy was the dominant cost-effective decision in this set of patients. Patients treated with an antibiotic regimen that was in excess of the recommendations of the 2007 IDSA/ATS guidelines experienced lower costs and increased utility compared to patient treated with an adherent or under-treatment regimen. On average, total hospitalization costs were $13,854 and $3461 more in the adherent group relative to the over- and under-treated groups, respectively.

**Table 5:** Cost effectiveness results in the ICU model of hospitalized community-acquired pneumonia in the elderly according to antibiotic regimen. Increment cost, utility, and cost/utility ratio were estimated relative to adherent as the base comparator

	Adherent	Over-Treated	Under-Treated
Cost ($)	44,765 (20,243 – 76,890)	30,912 (11,383 – 60,682)	41,305 (17,102 – 74,067)
Incremental Cost ($)		−13,854 (−51,699 – 24,938)	−3461 (−44,741 – 37,677)
Utility	7.3 (4.3 – 10.6)	9.6 (5.2 – 12.3)	7.5 (3.9 – 11.1)
Incremental Utility		+2.3 (−3.3 – 6.8)	+0.2 (−8.8 – 5.2)

Estimates presented as means (2.5^th^-97.5^th^ percentile). Costs are in 2013 US dollars

### Sensitivity analyses

Sensitivity analyses are summarized in Tables 6 and 7. The over-treatment strategy remained the dominant strategy across all of the ranges in the sensitivity analyses for drug cost estimates in the ICU model. In the ward model, the adherent strategy was dominant across all variations in ward oral drug costs, but partially dominant across variations in ward intravenous drug costs.

**Table 6:** Sensitivity analysis of model parameters

	Dominant Strategy	
	ICU	Ward
ICU Intravenous Drugs^a^		
Adherent	Never Dominant	
Over-Treatment	Dominant	N/A
Under-Treatment	Never Dominant	
Ward Intravenous Drugs^a^		
Adherent	Never Dominant	Dominant when Under-Treatment IV drugs > $25
Over-Treatment	Dominant	Never Dominant
Under-Treatment	Never Dominant	Dominant in circumstances when Under-Treatment IV drugs < $25 and Adherent IV Drugs > $70
Ward Oral Drugs^a^		
Adherent	Never Dominant	Dominant
Over-Treatment	Dominant	Never Dominant
Under-Treatment	Never Dominant	Never Dominant

^a^ 3-Way analysis across daily drug costs of $1 to $100 in 20 intervals

**Table 7:** Sensitivity analysis of utility and cost estimates (fixed)

	Dominant Strategy	
	ICU	Ward
Daily Hospital Costs		
Adherent	Never Dominant	Dominant
Over-Treatment	Dominant when daily ICU costs > $2675	Never Dominant
Under-Treatment	Partially dominant when daily ICU costs < $2675 and daily ward costs > $835^a^	Never Dominant^b^
Utility Estimates		
Adherent	Never Dominant	Dominant
Over-Treatment	Dominant	Never Dominant
Under-Treatment	Never Dominant^c^	Never Dominant^d^

^a^ Two-way analysis, $100 to $5000 in 20 intervals (ward) and $500 to $15,000 in 20 intervals (ICU)

^b^ One-way analysis, $100 to $5000 in 20 intervals

^c^ 3-way analysis, 0.1 to 0.9 in 10 intervals for utility estimates (ICU, clinically stable, and ward)

^d^ Two-way analysis, 0.1 to 0.9 in 10 intervals for utility estimates (clinically stable and ward)

**Fig. 5:**
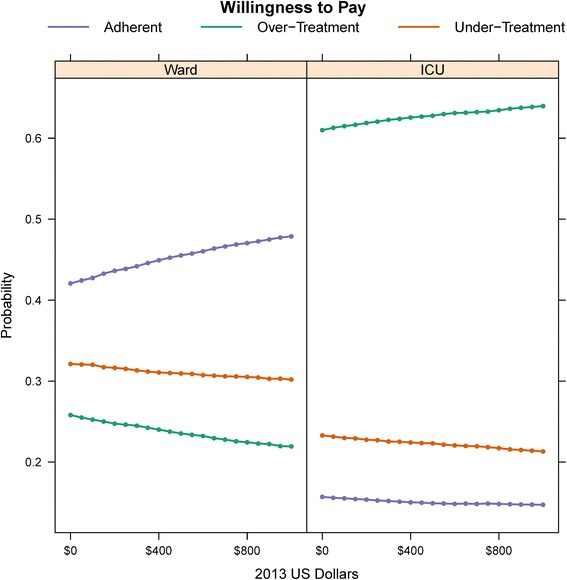
Dominant strategy proportions by willingness to pay. The proportion of times a given antibiotic regimen is dominant according to different willingness to pay thresholds, stratified by ICU or ward admission status

We determined changes in the dominant strategy proportion over a range of willingness to pay values in the probabilistic sensitivity analysis. Since the model measured daily cost and utility estimates, a conversion factor was used and we varied the willingness to pay across a range from 0 to $1000 per quality-adjusted life day, corresponding to 0 to $365,000 per QALY [24]. The over-treatment strategy remained the dominant strategy in > 60 % of the iterations in the ICU model across all levels of willingness to pay, while the adherent strategy remained the dominant strategy in the Ward model (Fig. 5).

Differences in length of stay were the most important determinants of cost in the models, particularly in the ICU. The dominant strategy of over-treatment in the ICU was associated with lower overall length of stay (median = 11 days) compared to the adherent and under-treatment strategy (median = 14 days for both). ICU length of stay varied greatly for the three strategies: over-treatment (median = 4 days), adherent (median = 12 days), and under-treatment (median = 8 days). Length of stay did not vary quite as much in the ward model: adherent (median = 8 days), over-treatment (median = 10 days), and under-treatment (median = 9 days).

## Discussion

The most important finding in this work is that adherence to the IDSA/ATS guidelines regarding empiric antibiotic therapy for elderly patients hospitalized with CAP was cost effective compared to non-adherence in non-ICU patients, but was not the most cost effective strategy in ICU patients. Elderly patients admitted to the ICU with CAP who were treated with an antibiotic regimen in excess of the IDSA/ATS guidelines had lower costs and increased quality of life compared to those treated with an adherent regimen.

The cost effectiveness implications of adherence to treatment guidelines has been evaluated in a limited number of settings, finding that in general, adherence to treatment guidelines is cost effective. Adherence with guidelines for sarcoma treatment was cost effective in a recent study from two European regions [12]. Adherence to guidelines has also been shown to be cost-effective in such diverse fields as intrauterine insemination and hepatitis B therapy [10, 11]. A study from Japan has shown the adherence to guidelines for gastric ulcer therapy is cost effective in that country [9]. Consensus treatment and practice guidelines have the potential to not only standardize care and disseminate best practice measures throughout complex healthcare systems, but also may offer cost effective strategies that are of particular importance in the current era of health care utilization reform.

Adherence to guidelines for the treatment of CAP has been associated with improved outcomes. An analysis of the same CAPO data used in this study found adherence to guidelines improved mortality, LOS, and time to clinical stability in elderly patients hospitalized with CAP [8]. However, the previous study did not separate patients on the basis of admission to the ICU or ward. Nonadherence to guidelines was associated with in-hospital mortality in studies of both community hospitals and tertiary teaching hospitals [25, 26]. These findings of improved outcomes in both hospitalized and outpatient settings with adherence to antibiotic guidelines have been replicated [27–29]. Nonadherence rates were higher in patients treated by non-pulmonary specialists in one study [30]. The cost implications of adherence to guidelines in hospitalized patients with CAP have not been studied as extensively. One study from Europe evaluated the cost-effectiveness of adherence to Spanish guidelines for the treatment of hospitalized CAP in non-ICU patients of any age [13]. This study found that adherence to the guidelines was the dominant cost-effective strategy compared to non-adherence. To our knowledge, this study is the first to evaluate the cost-effectiveness of adherence to guidelines in an exclusively elderly population with CAP in a multi-institutional setting, stratified by admission to the ICU or ward. Additionally, this study accounts for the stochastic nature of a hospital stay by estimating conditional transition probabilities directly from the data using the Aalen-Johansen estimator. This is an improved method by which to estimate cost and utility changes during a hospitalization course, as one is able to estimate daily, conditional changes in the probability of transitioning between states rather than averaging the transition probabilities over the course of an entire hospitalization, thus providing more precise estimates of cost and utility changes.

While the finding that adherence to IDSA/ATS guidelines was the dominant strategy in ward (non-ICU) patients was expected and is consistent with a previous report, it was surprising to find that over-treatment was the dominant strategy in ICU patients [13]. A close analysis of the state occupation probabilities used to build the model suggests the likely explanation is the accelerated time to clinical stability in this cohort of patients, which under our assumptions led to a reduction in ICU LOS for these patients. Daily ICU costs were the dominant driver of total costs in this model. Drug costs, which are a direct consequence of empiric antibiotic therapy choice, are miniscule compared to the daily cost of a stay in the ICU, which is measured in thousands of dollars per day. Thus, any large cost benefit from an antibiotic choice in relationship with the IDSA/ATS guidelines would likely be driven by a reduction in ICU and overall LOS rather than a reduction in daily drug costs. In the CAPO cohort, ICU LOS was shorter in patients who were treated with an over-treated antibiotic regimen compared to an adherent strategy. By hospital day 5, only 43 % of over-treated patients initially admitted to the ICU remained there, while the percentage of patients treated with an adherent regimen remaining in the ICU was nearly twice that (71 %) (Fig. 2). This led to dramatic reductions in daily and cumulative costs, and subsequent improvements in utility for the over-treated group. The marginal (unadjusted) 14-day mortality rate was higher in the over-treated group (18 %) compared to the adherent group (14 %) (Table 1), though differences in adjusted mortality rates were smaller (4.1 % vs. 2.4 %, respectively, Fig. 2). Overall, the lower costs associated with quicker transfer from the ICU to the ward made the over-adherent treatment strategy the most cost effective.

From this analysis, one cannot draw a conclusion of direct causation regarding the implementation of an overly-broad antibiotic regimen and the reduction in ICU LOS, although there certainly seems to be a strong association between an over-treatment strategy and a decreased ICU LOS that leads to reduced hospital costs. One potential explanation is the possibility that the patients in the over-treatment cohort were over-triaged to the ICU compared to adherent and under-treated patients and thus were more likely to leave the ICU earlier, independent of initial empiric antibiotic coverage. However, the initial demographics of our ICU patients suggest that the three antibiotic groups were relatively similar (Table 1). Pulmonary severity indices were not significantly different across the antibiotic groups, nor were the differences in comorbid conditions such as cancer, congestive heart failure, COPD, renal, or liver disease. Though the sample size for the ICU was small, over-treated patients did have a significantly higher probability to reach clinical stability by day 7 (Table 1), and in particular patients that received both a macrolide and a quinolone in addition to a β-lactam. Interestingly, another recent study of hospitalized CAP patients from three world regions found an elevated risk of mortality associated with ICU patients prescribed flouroquinolones relative to macrolides [31]. However, this study did not investigate whether the combination of a flouroquinolone with a macrolide provided any additional benefit, nor did it investigate time to clinical stability or length of hospital stay. These findings suggest that strategies to reduce ICU LOS may include aggressive empiric antibiotic regimens that can be later tailored to more targeted therapy guided by clinical response and microbial laboratory findings.

The findings in this study were robust over a wide range of sensitivity analyses performed. Sensitivity analyses of model parameters in multi-state Markov models are a critical component of the evaluation of model findings, as the model predictions are only as good as the parameters used to build the model. Antibiotic costs were estimated from a single local institution (University of Louisville Hospital), thus a valid criticism would be the external validity of these cost estimates to other hospitals. Sensitivity analyses found that the dominant strategies were robust over a wide range of daily antibiotic costs, thus suggesting that these findings have external validity to other institutions. Utility estimates are also quite subjective. We attempted to control for this using an expert panel of CAPO investigators who are familiar with the care of elderly patients with CAP. Modeling the utility estimates as a probability helps to account for this variability in the probabilistic sensitivity analysis. State occupation probabilities were available for the entire 30 day hospital course for our study cohort. We limited our model to an analysis of only the first 14 days, reasoning that hospitalizations beyond 14 days for CAP in the elderly likely were the result of complex, confounding factors and associated comorbidities that were unaffected by the initial empiric antibiotic coverage. Sensitivity analysis, however, did confirm the cost dominant strategies remained the same for models analyzed > 14 days.

The results reported here are based on the adjusted transition probability estimates based on the parametric models. However, conclusions based on the non-parametric estimates were similar, with adherence being the dominant strategy for the ward and over-treated the dominant strategy for the ICU. If the setting of the Markov model was extended to include post-discharge utility, then the difference in unadjusted mortality between the adherent and other antiobiotic groups might have a greater impact on the cost-effectiveness analysis. The most salient difference between the adjusted and unadjusted models was the reduced probability of mortality and increased probability of discharge and reaching clinical stability in the ICU for the adjusted model relative to the unadjusted estimates (Additional file 5).

The findings in this study must be considered with consideration of the shortcomings. Decision analysis models are only as strong as the parameters used to build them. While every effort was made to develop the model with realistic parameters, limitations arise in estimating transition probabilities, cost and utility estimates, and perspective of the model. Baseline differences in the clinical risk factors among the three groups, including PSI score and age, clearly have an impact on estimates of in-hospital mortality and discharge rates. While attempts were made to limit their effect on the model using adjusted transition probability estimates, differences in clinical pathways, practice, and resource utilization among the participating institutions and their effects on the clinical outcomes measured in this study cannot be discerned. An additional limitation is the assumption that patients transitioned out of the ICU once they reached clinical stability. This may not necessarily be the case, as patients may require longer stays due to deterioration of other comorbidities or other reasons. However, on average patients who reach clinical stability sooner should also leave the ICU earlier, so the observed differences between antibiotic treatment classes should be relatively robust. Finally, the estimated transition probabilities for over-treated patients in the ICU were based on only 28 patients, and require independent validation of the finding that this treatment regime was most cost-effective for these patients in the hospital setting.

A second limitation of the model is how patient utility estimates were obtained. The sample size of expert opinions (six) was limited and only from US institutions. While the experts were independently polled and we have no reason to believe there responses were subject to bias, an alternative method for obtaining a consensus (e.g., the Delphi method) might be preferable. Co-morbidities were not factored into the utility estimates both to simplify the data collection and because we felt this was not directly related to CAP/CAP therapy. Finally, using expert opinions to model patient-centric utilities is less desirable than patient-based estimates. While expert judgments concerning utilities should be used sparingly, it became pragmatically difficult to elicit utilities from patients and a data source does not exist in which the specific utilities we needed were present. A superior approach would be to estimate utilities based on appropriate patient questionnaires, perhaps as part of a prospective study to include post-discharge outcomes. While we did not have the resources to obtain such data as part of this study, future research will definitely take this into consideration.

Third, the CAPO data used in this study does not contain cost data, and external references and data were used to model both hospitalization and antibiotic regimen costs. While it may prima facie seem inconsistent to model costs from two different sources, we elected to use data from Dasta et al. [22] in addition to Kaplan et al. [23] for two reasons. First, the data from Dasta et al. suggests that ICU costs are not constant, rather they change over time. Hence these data are uniquely appropriate for use in a Markov model, in which one can capture these differences and make more accurate inferences about differences in costs based on how many days are spent in the ICU. Second, the data from Kaplan et al. only provides estimates of the total hospitalization costs for patients initially treated for CAP in the ICU. Thus these estimates capture both costs in the ICU and in the ward, and an average cost per day calculation from the Kaplan data would be “contaminated” with time spent on the ward. Hence it is a less fit estimate for daily ICU costs compared to the Dasta et al. data. Lastly, costs were estimated based on US institutional rates but applied to international data. However, since the results were consistent across sensitivity analyses for costs and willingness to pay thresholds, we feel the conclusions are relatively robust to any regional variation in costs.

Finally, the conclusions here are based on the short-term utility and cost-effectiveness for CAP patients during their hospital stay. Since we did not have direct data concerning post-discharge mortality and quality of life for this cohort of patients, we did not attempt to model long-term cost-effectiveness. While we have no evidence to suggest that post-hospitalization outcomes are different among the treatment cohorts in this study, we acknowledge that it is a limitation of the model that it incorporates only a fraction of the life-expectancy for these patients. However, our multi-state modeling approach is easily extendable to prospectively collected data on patients discharged after hospitalization due to CAP [32–35]. For example, one could model the post-discharge outcomes as additional states within the Markov model, tracking transitions between occurrence of various co-morbidities, re-hospitalizations, and mortality. The multi-state model can also be coupled with causal inference approaches to assess the effect of various interventions/treatment regimens on outcomes of interest. For example, such models have been fit in other disciplines to evaluate whether partial versus full time sick leave and having a cooperation agreement is helpful in reducing sickness absence from work [36]. In a similar fashion, a multi-state model coupled with causal inference approaches may form the basis of active, prospective evaluation of antibiotic choices and the cost-effectiveness of adherence to recommended guidelines. Patient quality of life would then be ideally collected using appropriate designed patient questionnaires. As a final thought, such studies would also need to take into consideration the effect of antibiotic regimen on antimicrobial resistance. The societal costs of antibiotic over-treatment pressures on the development of antimicrobial resistance are hidden costs to the over-treatment strategy that must be considered.

## Conclusion

In conclusion, this study found the adherence to IDSA/ATS guidelines in elderly patients hospitalized with CAP was cost-effective for patients admitted to a non-ICU setting. For patients admitted to the ICU, over-treatment relative to adherence to IDSA/ATS guidelines was associated with decreased ICU and overall LOS; subsequent to these LOS reductions, over-treatment was the most cost-effective strategy in these particular patients. The prospective evaluation of empiric antibiotic treatments in CAP and the resulting cost implications of these choices remain to be determined.

## Availability of data and materials

The CAPO database is owned and maintained by the Division of Infectious Diseases at the University of Louisville (see http://caposite.com). Permission was granted to access the data by Dr. Julio Ramirez. The specific data used in this study is available upon request from the authors.

## Additional files

Additional file 1: Daily antibiotic cost estimates, based on average wholesale prices (year 2013 value) of each antibiotic obtained from University of Louisville Hospital. (PDF 41.8 kb) Additional file 2: A copy of the survey questionnaire given to the expert panel regarding patient utilities. (PDF 8.28 kb) Additional file 3: Daily transition probabilities for adherent, under-treated, and overtreated patients admitted to the ward for CAP. Solid lines represent adjusted probabilities based on the Cox regression model using the most common values of the covariates (see description in text), while dashed lines represent non-parametric estimates. (PDF 7.51 kb) Additional file 4: Daily transition probabilities for adherent, under-treated, and overtreated patients admitted to the ICU for CAP. Solid lines represent adjusted probabilities based on the Cox regression model using the most common values of the covariates (see description in text), while dashed lines represent nonparametric estimates. (PDF 7.16 kb) Additional file 5: State occupation probabilities (SOPs) for adherent, under-treated, and over-treated patients admitted to the ICU for CAP. Solid lines represent adjusted probabilities based on the Cox regression model using the most common values of the covariates (see description in text), while dashed lines represent non-parametric estimates. (PDF 7.06 kb)
