# Supplementary material for: Cost effectiveness of adherence to IDSA/ATS guidelines in elderly patients hospitalized for Community-Aquired Pneumonia
Source: BMC Med Inform Decis Mak. 2016 Mar 15;16:34. doi: 10.1186/s12911-016-0270-y (PMC4791973; doi:10.1186/s12911-016-0270-y)
Supplement: Additional file 1: — Daily antibiotic cost estimates, based on average wholesale prices (year 2013 value) of each antibiotic obtained from University of Louisville Hospital. (PDF 41.8 kb) [file 12911_2016_270_MOESM1_ESM.pdf]

Additional File 1. Daily antibiotic cost estimates

Antibiotic	Cost ($) per dose	Avg # doses per day	Cost ($) per treatment day
Azithromycin 500mg IV vial	7.20	1	7.20
250mg tablet	7.40	1	7.40
Ceftriaxone 1g IV vial	46.00	1	46.00
Imipenem/Cilastin 500mg IV vial	23.52	4	94.08
Piperacillin/Tazobactam 3.375g IV vial	6.50	4	26.00
Ceftazidime 2g IV vial	19.97	3	59.91
Cefotaxime 1g IV vial	6.60	3	19.80
Cefepime 1g IV vial	20.33	3	60.99
Amoxicillin/clavulanate 875/125 tablet	4.90	2	9.80
Cefuroxime 1.5g IV vial	5.40	2	10.80
250mg tablet	4.40	2	8.80
Ticarcillin/Clavulanic 3.1g IV vial	10.60	4	42.40
Ampicillin/Sulbactam 3g IV vial	6.36	4	25.44
Clindamycin 600mg IV dose	1.87	3	5.61
300mg tablet	3.72	3	11.61
Metronidazole 500mg IV bag	1.33	3	3.99
500mg tablet	0.73	3	2.19
Cefazolin 1g IV premixed bag	5.04	3	15.12
Piperacillin 4g vial	16.70	4	66.80
Vancomycin IV 1g vial	7.50	2	15.00
Clarithromycin 500mg tablet	6.13	2	12.26
Levofloxacin 750mg IV bag	13.20	1	13.20
750mg tablet	19.74	1	19.74
Ciprofloxacin 400mg IV bag	3.19	2	6.38
500mg tablet	3.17	2	6.34
Gentamicin 120mg IV dose	7.41	3	22.23
Ampicillin IV 500mg IV vial	2.90	4	11.60
Penicillin G = Penicillin 5MU vial	8.70	6	52.20
Trim/Sulfameth DS tablet PO	1.08	2	2.16
Meropenem 500mg IV vial	35.19	4	140.76
Moxifloxacin 400mg bag	42.00	1	42.00
400mg tablet	22.80	1	22.80
Erythromycin PO 250mg tablet	2.50	4	10.00
Chloramphenicol 1g IV vial	35.90	4	143.60
Aztreonam 1g IV vial	34.80	3	104.40
Rifampin 600mg IV vial	78.00	1	78.00
150mg cap x4 caps	6.32	1	6.32
