# Supplementary material for: Cost effectiveness of adherence to IDSA/ATS guidelines in elderly patients hospitalized for Community-Aquired Pneumonia
Source: BMC Med Inform Decis Mak. 2016 Mar 15;16:34. doi: 10.1186/s12911-016-0270-y (PMC4791973; doi:10.1186/s12911-016-0270-y)
Supplement: Additional file 2: — A copy of the survey questionnaire given to the expert panel regarding patient utilities. (PDF 8.28 kb) [file 12911_2016_270_MOESM2_ESM.pdf]

Additional File 2. Expert panel survey questionnaire

1) In what country to you practice? _______________________________

2) What is your specialty? Internal Medicine

Infectious Disease

Pulmonology / Critical Care

Other

3) What is your academic rank? Fellow/Resident

Assistant Professor

Associate Professor

Professor

4) For how many years have you been practicing medicine since medical school?_______

4) What is your age? ___________

5) What is your gender? Male Female

Please assign your estimated utility, on a scale from 0 to 1, for the average patient age > 65 years old who is hospitalized for community-acquired pneumonia in the following condition:

Admitted to the ICU ___________

Admitted to the ward, but not well enough to be considered clinically stable________

Admitted to the ward, considered to be clinically stable___________
