# Supplementary material for: Cost effectiveness of adherence to IDSA/ATS guidelines in elderly patients hospitalized for Community-Aquired Pneumonia
Source: BMC Med Inform Decis Mak. 2016 Mar 15;16:34. doi: 10.1186/s12911-016-0270-y (PMC4791973; doi:10.1186/s12911-016-0270-y)
Supplement: Additional file 4: — Daily transition probabilities for adherent, under-treated, and overtreated patients admitted to the ICU for CAP. Solid lines represent adjusted probabilities based on the Cox regression model using the most common values of the covariates (see description in text), while dashed lines represent nonparametric estimates. (PDF 7.16 kb) [file 12911_2016_270_MOESM4_ESM.pdf]

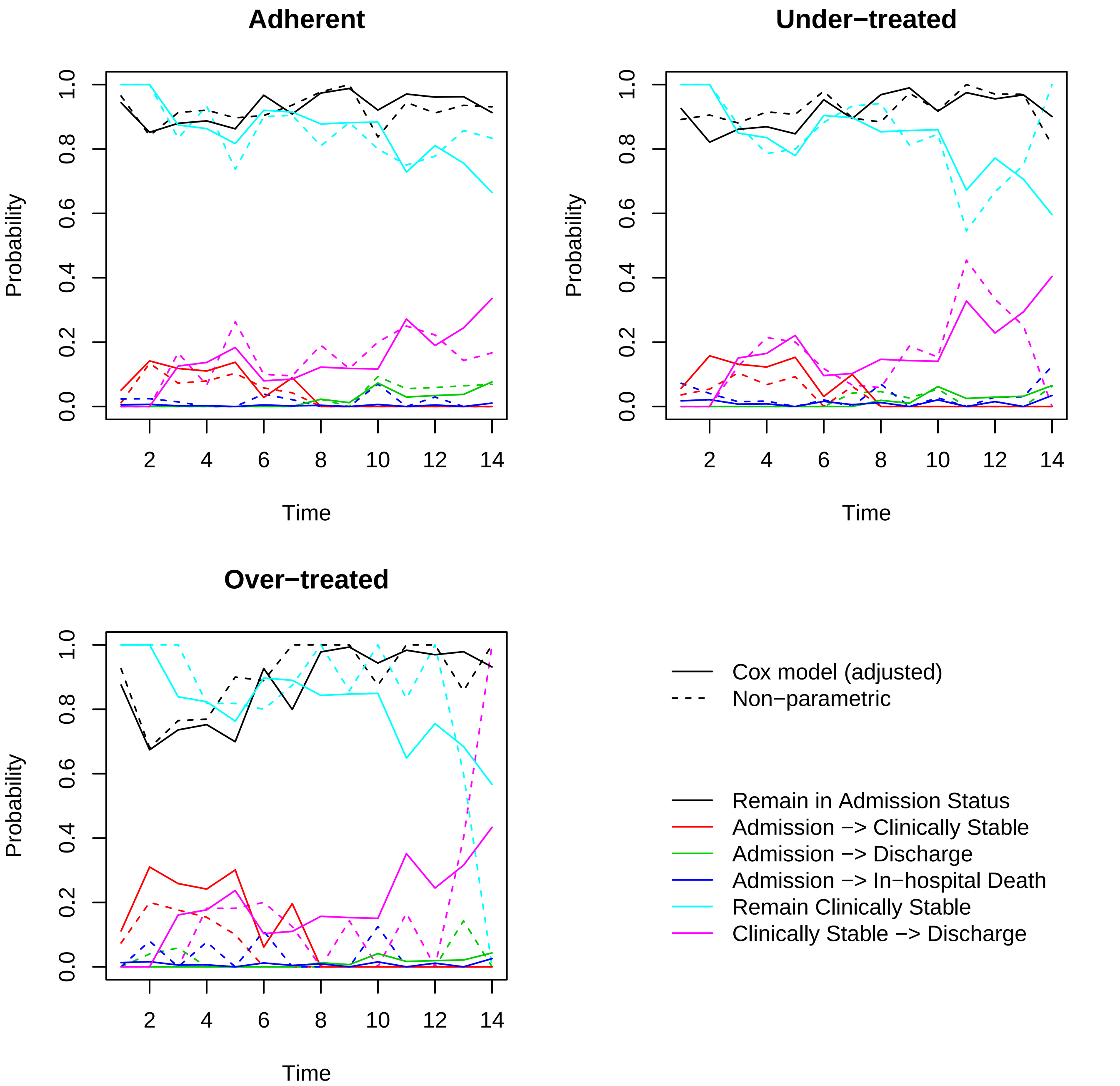
Adherent
Under−treated
Over−treated
Probability
Time
0.0
0.2
0.4
0.6
0.8
1.0
2
4
6
8
10
12
14
Cox model (adjusted)
Non−parametric
Remain in Admission Status
Admission −> Clinically Stable
Admission −> Discharge
Admission −> In−hospital Death
Remain Clinically Stable
Clinically Stable −> Discharge
