# Supplementary material for: Cost effectiveness of adherence to IDSA/ATS guidelines in elderly patients hospitalized for Community-Aquired Pneumonia
Source: BMC Med Inform Decis Mak. 2016 Mar 15;16:34. doi: 10.1186/s12911-016-0270-y (PMC4791973; doi:10.1186/s12911-016-0270-y)
Supplement: Additional file 5: — State occupation probabilities (SOPs) for adherent, under-treated, and over-treated patients admitted to the ICU for CAP. Solid lines represent adjusted probabilities based on the Cox regression model using the most common values of the covariates (see description in text), while dashed lines represent non-parametric estimates. (PDF 7.06 kb) [file 12911_2016_270_MOESM5_ESM.pdf]

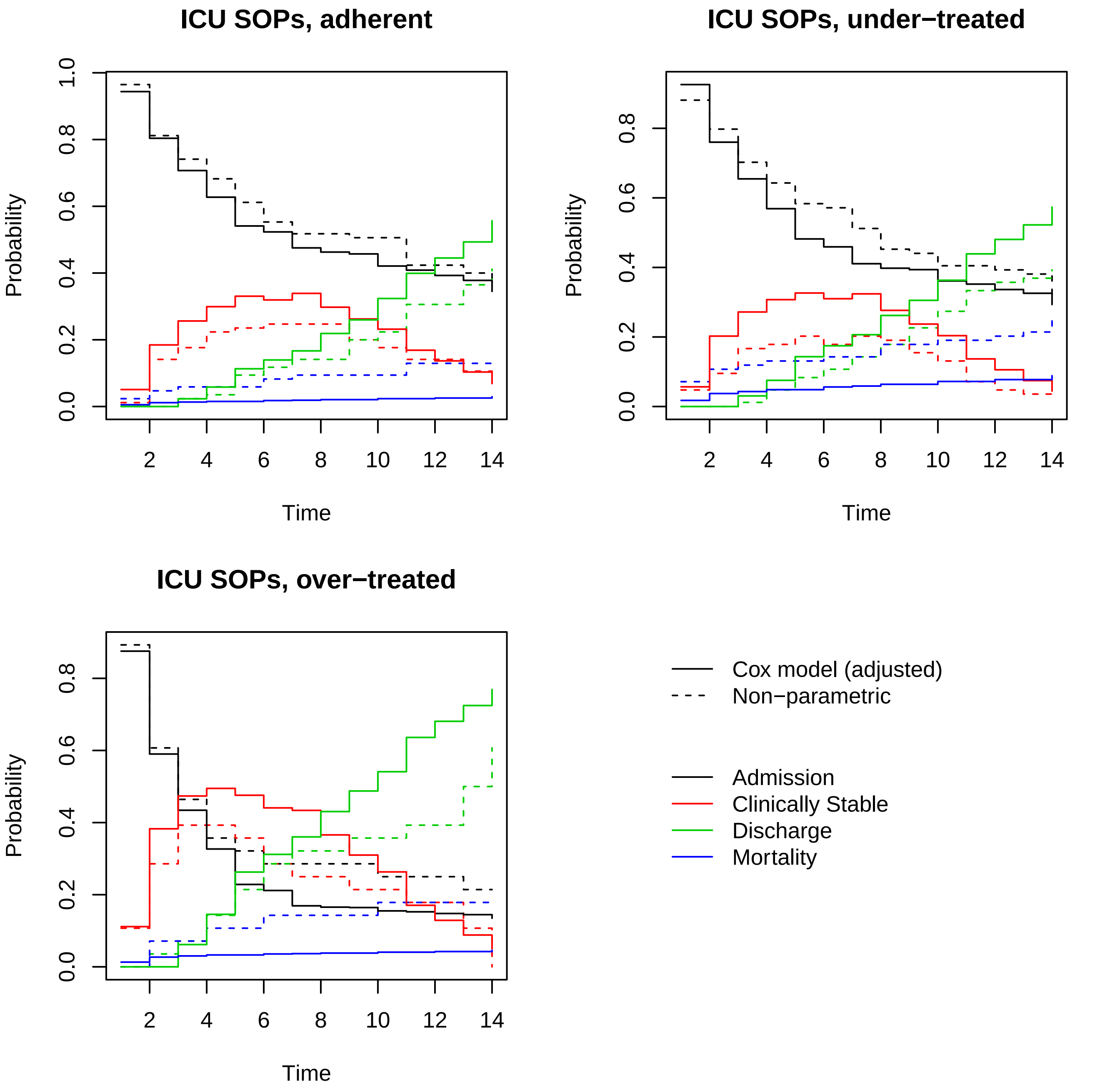

ICU SOPs, adherent
ICU SOPs, under−treated
ICU SOPs, over−treated
Probability
Time
0.0
0.2
0.4
0.6
0.8
1.0
2
4
6
8
10
12
14
Cox model (adjusted)
Non−parametric
Admission
Clinically Stable
Discharge
Mortality
